# Enhancing dementia diagnosis data in UK Biobank: the UK Biobank Brain Health Study

**DOI:** 10.1002/alz70860_106885

**Published:** 2025-12-23

**Authors:** Janet Maccora, Jo Holliday, Elizabeth Taylor, Jelena Bešević, Christopher Stibbards, Gemma F Codner, Martin K Rutter, Adam J Lewandowski, Naomi Allen

**Affiliations:** ^1^ University of Oxford, Oxford, Oxfordshire, United Kingdom; ^2^ UK Biobank Ltd, Stockport, Greater Manchester, United Kingdom; ^3^ Manchester University NHS Foundation Trust, Manchester, Greater Manchester, United Kingdom; ^4^ University of Manchester, Manchester, Greater Manchester, United Kingdom

## Abstract

**Background:**

As the UK Biobank population of half a million participants enters older age (current mean age 73 years), the number of dementia cases in the cohort is rapidly increasing. At present, individuals living with dementia can be identified through linkage to electronic hospital and death records, however these diagnoses are poorly characterized in terms of dementia subtypes and progressive stages. This limits the potential for robust and innovative therapeutic and preventative treatment approaches to be developed. The aim of the UK Biobank Brain Health Study is to enhance the characterization of neurodegenerative diseases and their subtypes by collecting new data on participants with a range of neurodegenerative symptoms or diagnoses.

**Method:**

Experts across a diverse range of settings – including academic, clinical, operational and experiential – were consulted to provide input to the study design. Information was gathered via domain‐specific working groups, literature reviews, online surveys, site visits, facilitated workshops, and oral and written feedback.

**Result:**

Several challenges arose during the study design phase, including how to identify UK Biobank participants with neurodegenerative disease, how to contact and recruit these participants who may find it difficult to engage, how to plan a study visit that is sensitive to the needs of people living with neurological impairment, and how to administer standardized assessments at scale across multiple locations. Nevertheless, extensive internal and external collaboration facilitated the development of a pilot study protocol involving MRI brain imaging, cognitive testing, blood assays, and remote activity and sleep assessment in 60‐100 participants.

**Conclusion:**

This pilot phase of the Brain Health Study will test the feasibility and acceptability of the study protocol and inform the main phase study design. Although numerous obstacles to enriching dementia diagnosis data in large biobank studies were identified during the planning stage, the Brain Health Study attempts to mitigate major barriers by listening to, and acting on, the breadth of experience and advice available from experts including participant‐facing staff, participant advisory groups, and people living with dementia and their families.

